# Ischemic heart disease mortality with documented tobacco use in the United States, 1999–2023: a nationwide population-based analysis

**DOI:** 10.1186/s13690-026-02013-y

**Published:** 2026-07-18

**Authors:** Alyaa Ahmed Ibrahim, Zuha Tariq, Abdul Samad, Ahmed Khairy, Alaa Eldeeb, Maha Sajjad, Abdelrhman H. Mohamed, Walid Bechibchi, Mohamed Alatfawy, Karim Othman Abdelnaim, Amr Ibrahim, Ahmed Atef Mohamed, Rodina Sharaf, Irfan Ullah, Mohamed Fawzi Hemida

**Affiliations:** 1 Faculty of Medicine, Alexandria University, Alexandria, Egypt; 2 Department of Medicine, Allama Iqbal Medical College, Lahore, Pakistan; 3 Department of Medicine, Dow University of Health Sciences, Karachi, Pakistan; 4 Department of Medicine, King Edward Medical University, Lahore, Pakistan; 5 Faculty of Medicine, Luxor University, Luxor, Egypt; 6 Faculty of Medicine, University of Constantine 3, Salah Boubnider, Constantine, Algeria; 7 Faculty of Medicine, Kafr El-Sheikh University, Kafr El-Sheikh, Egypt; 8 Faculty of Medicine, Ain Shams University, Cairo, Egypt; 9 Faculty of Medicine, Monoufia University, Monoufia, Egypt; 10 Faculty of Medicine, Helwan University, Cairo, Egypt; 11 Department of Cardiovascular Medicine, University Hospitals Harrington Heart and Vascular Institute, Cleveland, OH USA; 12 American Society for Inclusion, Diversity, and Equity in Healthcare (ASIDE), Delaware, USA

**Keywords:** Tobacco use, Ischemic‍‌ heart disease, Mortality, Cardiovascular disease

## Abstract

**Background:**

Tobacco use is a major risk factor for ischemic heart disease (IHD), substantially increasing the likelihood of myocardial infarction, stroke, and premature mortality. This study examined U.S. trends in IHD mortality with documented tobacco use disorder from 1999 to 2023.

**Methods:**

CDC mortality data for adults aged ≥ 25 years with IHD (ICD-10 codes: I20–I25) and documented tobacco use (ICD-10 code: F17) listed as contributing causes of death were analyzed. Age-adjusted mortality rates (AAMRs) per 100,000 population were calculated. Joinpoint regression was used to estimate annual percent change (APC) and average annual percent change (AAPC).

**Results:**

From 1999 to 2023, 1,497,919 deaths were reported. The AAMR rose from 3.90 to 32.37 (AAPC: 10.71; *p* < 0.01). Males had a higher AAMR (39.68; AAPC: 10.72) than females (14.05; AAPC: 10.77). Non-Hispanic (NH) Whites had the highest AAMR (26.86), followed by American Indians (25.43), NH Blacks (19.77), and Hispanics (10.38). The Midwest had the highest regional AAMR (32.96). Most deaths occurred in inpatient facilities (27.9%). Adults aged ≥ 65 years had the highest mortality rate (CMR: 92.16). Rural areas exceeded urban (35.73 vs. 22.95).

**Conclusion:**

IHD mortality with documented tobacco use has risen sharply, disproportionately affecting older adults, males, rural areas, the Midwest, and NH Whites.

**Supplementary Information:**

The online version contains supplementary material available at 10.1186/s13690-026-02013-y.

**Table:** 

Text box 1. Contributions to the literature
● Provides one of the first long-term, nationwide analyses of mortality linked specifically to the combined burden of tobacco use and IHD in the United States. ● Demonstrates how IHD mortality with documented tobacco use has evolved over 25 years, offering updated surveillance evidence beyond traditional single-cause analyses.
● Informs tobacco control and cardiovascular prevention strategies by identifying high-risk populations that may benefit from targeted public health interventions.

## Introduction

Ischemic‍‌ heart disease (IHD) remains one of the major public health challenges and leading causes of mortality in the United States and worldwide. [1, 2] Despite the advances in prevention and treatment, IHD continues to account for a substantial proportion of preventable deaths. [3] It contributes significantly to the global cardiovascular mortality burden, with cardiovascular diseases (CVDs) collectively responsible for nearly one-third of all deaths worldwide. [4]

Among various risk factors for IHD, tobacco smoking is one of the most significant and modifiable factors [5]. Smoking is estimated to be the cause of approximately 1.62 million IHD deaths yearly which is around 18% of the total number of IHD-related deaths and thereby, it precipitates a significant burden of ill health, which is responsible for about 40.6 million Disability-adjusted Life-Years (DALYs) at the global ‍‌level. [6]

**Fig. 1:**
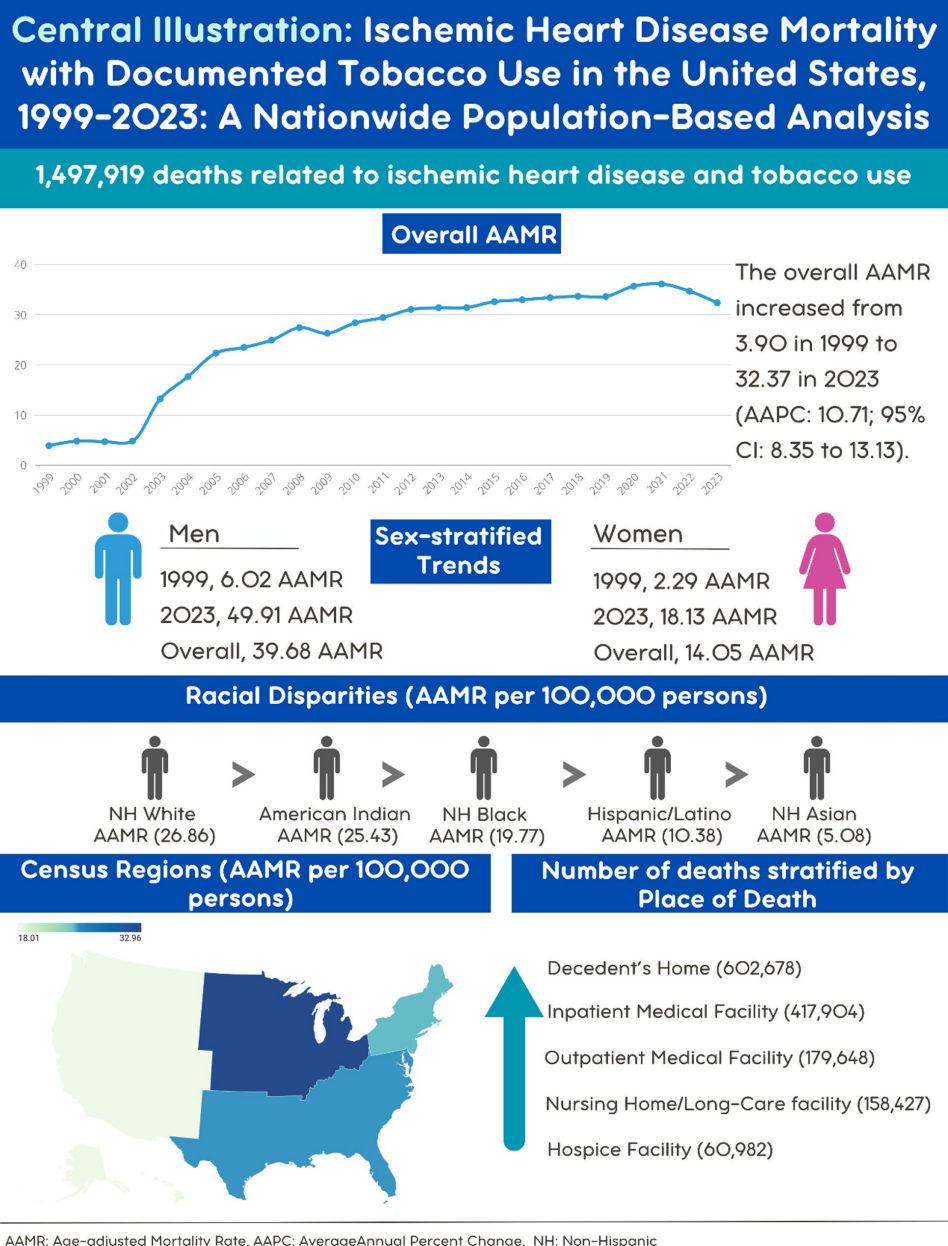
Central Illustration: Ischemic Heart Disease Mortality with Documented Tobacco Use in Adults Aged ≥ 25 years in the United States, 1999–2023: A Nationwide Population-Based

While‍‌ the portion of IHD caused by tobacco use has been reduced in over 80% of countries during the last 30 years, it is still a major challenge, especially in low- and middle-income countries as well as among older adults and men. [7] Although tobacco use is a well-established risk factor for IHD, national long-term mortality trends involving coexisting IHD and documented tobacco use disorder have been less extensively studied. As‍‌ a result, this research aims to explore the connection between tobacco use and IHD mortality trends in the United States through a nationwide population-based mortality analysis, which will provide updated data to guide the selection of the best prevention and intervention measures to alleviate the problem of ‍‌IHD with tobacco use as a contributing factor. (Figure 1)

## Methods

### Study design and population

A nationwide population-based mortality analysis was conducted using death certificate data retrieved from the Centers for Disease Control and Prevention Wide-Ranging Online Data for Epidemiologic Research (CDC WONDER) database. Data for adults aged 25 and older between 1999 and 2023 were analyzed to assess national mortality trends due to IHD and documented tobacco use disorder. This age cutoff has been used to define adults in a previous study [8]. Restricting to ≥ 25 improves robustness and reliability of estimates. We used the International Statistical Classification of Diseases and Related Health Problems-10th Revision (ICD-10) as follows: I20-I25 for IHD and F17 for documented tobacco use disorder and a published paper used the same codes for tobacco use [8]. Deaths were included if IHD and tobacco use disorder were listed anywhere on the death certificate, either as underlying causes or contributing causes of death. This comprehensive approach ensures the capture of all deaths where both conditions played documented roles, regardless of their position on the death certificate. Institutional review board approval was not required for this study as it used de-identified public use data provided by the government and adhered to the STROBE (Strengthening the Reporting of Observational Studies in Epidemiology) guidelines for reporting [9]. In accordance with CDC-WONDER database policies, death counts < 10 are suppressed to protect confidentiality. Consequently, suppressed data were treated as missing and excluded from analysis.

### Data abstraction

Data for population size, year, deaths, and demographics such as sex, age, race, region and states were extracted. Place of death was categorized into medical facilities, hospice, home and nursing home/long-term care facilities. Racial and ethnic categories were categorized into non-Hispanic (NH) White, NH Black or African American, NH American Indian or Alaskan Native, NH Asian or Pacific Islander, and Hispanic or Latino. The National Center for Health Statistics Urban-Rural Classification Scheme was used to assess the population by urban and rural counties per the 2013 U.S. census classification [10]. It is important to note that urban-rural data were consistently available and analyzed only for the period 1999–2020 due to historical limitations in CDC WONDERstratifications. Regions were stratified into Northeast, Midwest, South, and West according to the U.S. Census Bureau definitions. Age groups were divided into 3 groups: (25–44 ,45–64, and ≥ 65 years).

### Statistical analysis

Crude mortality rates (CMRs) and age adjusted mortality rates (AAMRs) per 100,000 population from 1999 to 2023 by year, sex, race/ethnicity, regions, and urban-rural status from 1999 to 2020 with 95% CIs were calculated, using the 2000 U.S. population as the standard [11]. CMRs were determined by dividing the number of individuals with IHD and documented tobacco use disorder by the corresponding U.S. population of that year. It’s worth mentioning that we used CMRs exclusively for age-specific analyses, as age adjustment would mask true differences between age groups. For all other variables, AAMRs were calculated to ensure comparability across populations with differing age structures. The Joinpoint Regression Program (Joinpoint V 5.4.0.0, National Cancer Institute) was used to determine the average annual percent change (AAPC) and the annual percent change (APC) with 95% CI in AAMR to quantify national annual mortality trends in IHD and tobacco use disorder. Joinpoint regression, a segmented regression technique, was used to identify points of trend change (join points) by fitting log-linear models and it used permutation tests to select the optimal number of join points, ensuring model fit [12]. This method allows identification of significant changes in AAMR over time by fitting log-linear regression models where temporal variation occurred. APCs were considered increasing or decreasing if the slope describing the change in mortality was significantly different from zero using two tailed t testing. A p value of < 0.05 was considered statistically significant. To account for potential temporal variation in ICD-10 F17 documentation practices, a sensitivity analysis restricted to deaths occurring from 2005 onwards was performed.

## Results

### Overall mortality trends

**Fig. 2:**
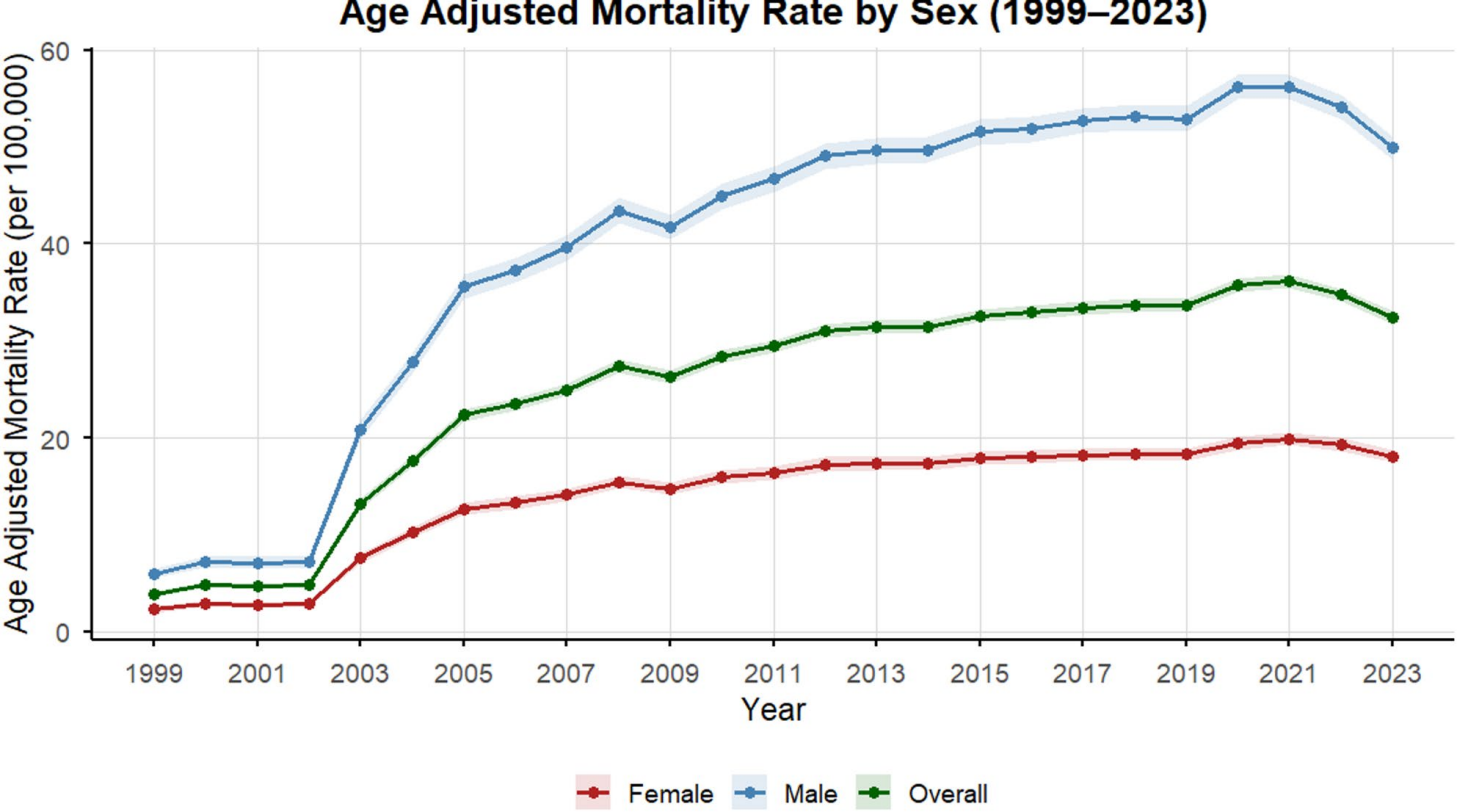
Ischemic Heart Disease Mortality with Documented Tobacco Use-Related Age-Adjusted Mortality Rates per 100,000, Stratified by Sex, in Adults Aged ≥ 25 years in the United States, 1999–2023. The line graph displays the temporal trends in mortality rates, comparing female, male, and overall adult populations over the 25-year study period

**Fig. 3:**
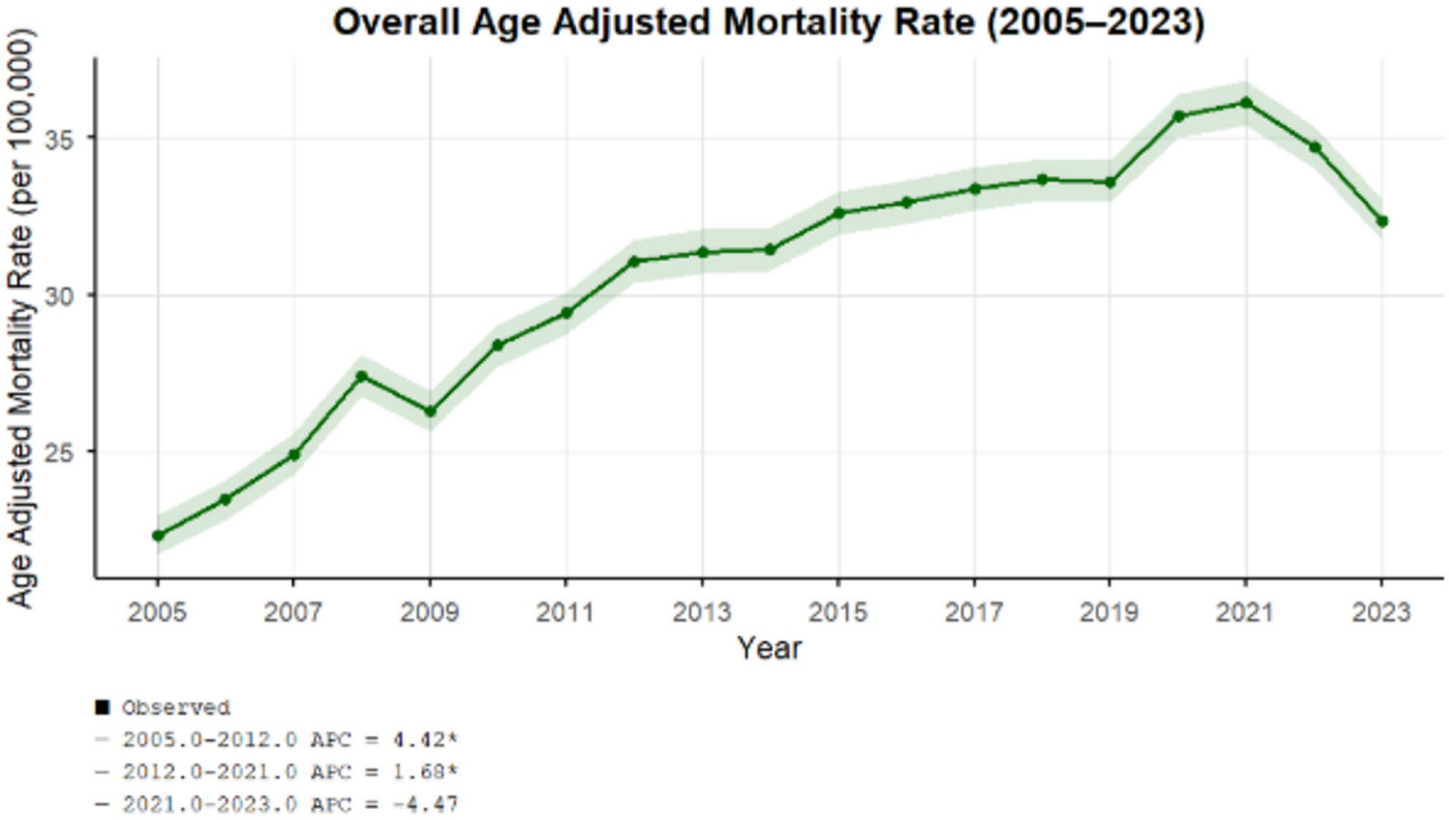
Sensitivity Analysis of Overall Ischemic Heart Disease Mortality with Documented Tobacco Use-Related Age-Adjusted Mortality Rates in Adults Aged ≥ 25 in the United States, 2005–2023. The graph illustrates the observed mortality rates along with the Annual Percent Change (APC) for distinct time segments: 2005–2012 (APC = 4.42*), 2012–2021(APC = 1.68*), and 2021–2023 (APC = -4.47)

Throughout the study period, IHD with documented tobacco use disorder contributed to the deaths of 1,497,919 individuals in the United States. The AAMR increased from 3.9 in 1999 to 32.37 in 2023 with an (AAPC of 10.71; 95% CI: 8.35 to 13.12; *p* < 0.01) with two joinpoints identified. A three phase upward trend was observed with mortality rate first increasing substantially from 3.9 in 1999 to 22.34 in 2005 with an (APC of 40.09; 95% CI: 29.38 to 51.67; *p* < 0.01), then there was a gentle rise from 22.34 in 2005 to 31.38 in 2013 with an (APC of 4.25; 95% CI: 1.09 to 7.51; *p* = 0.01), followed by a moderate incline from 31.38 in 2013 to 32.37 in 2023 with an (APC of 0.87; 95% CI: -0.61 to 2.38; *p* = 0.23) (Supplemental Tables 1–3; Fig. 2). Sensitivity analysis focusing on the 2005–2023 period corroborated these findings, demonstrating significant annual increases in mortality rates until 2021, followed by a recent downward trend through 2023 (APC = -4.47) (Fig. 3).

### Stratified by sex

Males exhibited higher AAMR (39.68) as compared to females (14.05), although both groups showed inclining trends. Among men, the AAMR increased from 6.02 in 1999 to 49.91 in 2023 with an (AAPC: 10.72; 95% CI: 8.39 to 13.10; *p* < 0.01) with two joinpoints identified, this incline was shown in three stages, at first, there was a considerable increase from 6.02 in 1999 to 35.59 in 2005 with an (APC: 41.33; 95% CI: 30.35 to 53.23; *p* < 0.01), then increasing from 35.59 to 51.54 in 2015 with an (APC: 3.61; 95% CI: 1.52 to 5.75; *p* = 0.02), after that, a slight decline was observed from 51.54 in 2015 to 49.91 in 2023 with an (APC: 0.19, 95% CI: -1.78 to 2.20; *p* = 0.85). In contrast, among women, the AAMR increased from 2.29 in 1999 to 18.13 in 2023 (AAPC: 10.77, 95% CI: 8.74 to 12.83; *p* < 0.01) with only one joinpoint identified, this increase was significant in two stages, first there was an increase from 2.29 in 1999 to 12.79 in 2005 with an (APC: 41.87, 95% CI: 31.51 to 53.06; *p* < 0.01), followed by gradual increase from 12.79 to 18.13 in 2023 with an (APC: 2.05, 95% CI: 1.32 to 2.68; *p* = 0.01) (Supplemental Tables 1–3; Fig. 2).

### Stratified by race

**Fig. 4:**
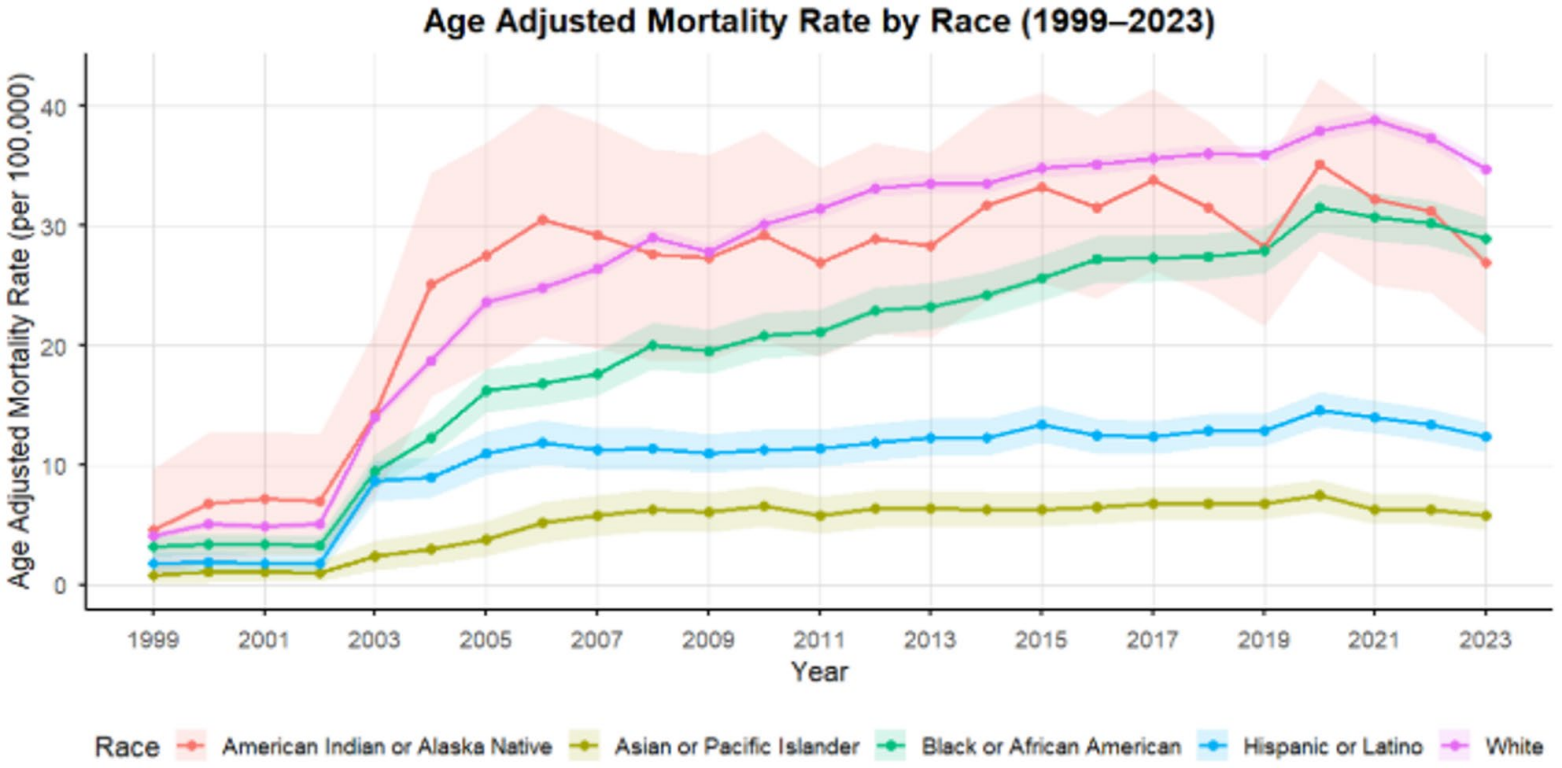
Ischemic Heart Disease Mortality with Documented Tobacco Use-Related Age-Adjusted Mortality Rates per 100,000, Stratified by Race, in Adults Aged ≥ 25 years in the United States, 1999–2023. Trends are categorized by race, including Non-Hispanic American Indian or Alaska Native, Non-Hispanic Asian or Pacific Islander, Non-Hispanic Black or African American, Non-Hispanic White, and Hispanic or Latino populations

The highest AAMR was observed in NH Whites (26.86), followed by American Indians or Alaska Natives (25.43), NH Black or African American (19.77), Hispanic population (10.38), and for Asian or Pacific Islander (5.08). Among NH Whites, the AAMR increased from 4.06 (95% CI: 3.96 to 4.16) in 1999 to 34.77 (95% CI: 34.53 to 35.02) in 2023 with an (AAPC: 10.66 (95% CI: 8.42 to 12.94; *p* < 0.01) with two joinpoints. This incline was notable between 1999 and 2005 increasing from 4.06 to 23.61 with an (APC of 39.45; 95% CI: 29.28 to 50.41; *p* < 0.01), and between 2005 and 2013 increasing from 23.61 to 33.53 with an (APC: 4.37; 95% CI: 1.42 to 7.42; *p* = 0.01), followed by a gentler rise through 2023 (APC: 0.93, 95% CI: -0.46 to 2.34; *p* = 0.18). For American Indian or Alaska Natives, the AAMR increased from 4.55 in 1999 to 26.9 in 2023 with an (AAPC: 8.95, 95% CI: 6.19 to 11.78; *p* < 0.01) with only one joinpoint identified, this increase was observed from 4.55 in 1999 to 27.49 in 2005 with an (APC: 38.73, 95% CI: 24.78 to 54.25; *p* = 0.03), and then there was a moderate decrease from 27.49 in 2005 to 26.9 in 2023 with an (APC: 0.52, 95% CI: -0.35 to 1.40; *p* = 0.23). The AAMR for NH Black or African American got increased from 3.16 in 1999 to 28.91 in 2023 with an (AAPC: 11.28, 95% CI: 8.90 to 13.71; *p* < 0.01) with two joinpoints. This incline occurred between 1999 and 2005 (APC: 39.45, 95% CI: 28.80 to 51.29; *p* < 0.01), and between 2005 and 2020 (APC: 4.19, 95% CI: 3.18 to 5.21; *p* < 0.01), followed by decrease from 2020 to 2023 (APC: -1.71, 95% CI: -9.23 to 6.44; *p* = 0.65) with two join points identified. Asian or Pacific Islander also exhibited a rise in overall mortality from 0.74 in 1999 to 5.75 in 2023 with an (AAPC: 9.70, 95% CI: 7.89 to 11.55; *p* < 0.01) with two joinpoints. First, mortality increased considerably from 0.74 in 1999 to 5.19 in 2006 with an (APC: 36.97, 95% CI: 29.86 to 44.48; *p* < 0.01), then there was a gentler rise from 5.19 in 2006 to 7.45 in 2020 with an (APC: 1.62, 95% CI: 0.84 to 2.39; *p* = 0.04), followed by a decline through 2023 (APC: -6.59, 95% CI: -12.22 to -0.59; *p* = 0.03). For Hispanic population, AAMR increased from 1.79 in 1999 to 12.37 in 2023 with an (AAPC: 10.78, 95% CI: 7.28 to 14.39; *p* < 0.01) with only one joinpoint, this increase was noticed between 1999 and 2004 (APC: 55.64, 95% CI: 32.52 to 82.79; *p* = 0.01) and between 2004 and 2023 (APC: 1.31, 95% CI: 0.47 to 2.13; *p* = 0.04) (Supplemental Tables 1, 2, 4; Fig. 4).

### Stratified by age

**Fig. 5:**
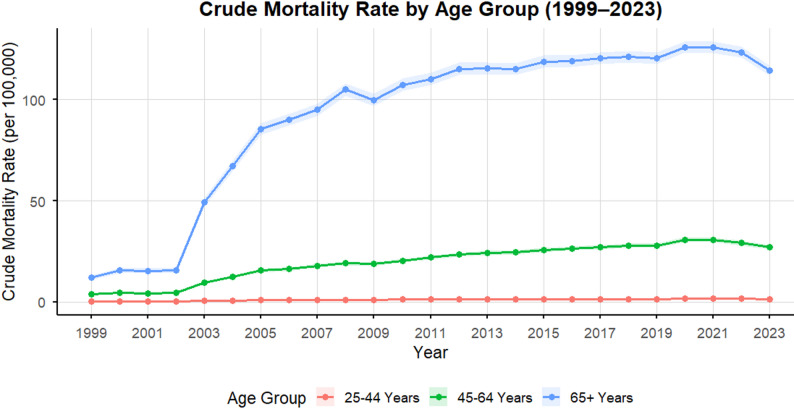
Ischemic Heart Disease Mortality with Documented Tobacco Use-Related Crude Mortality Rates per 100,000, Stratified by Age, in Adults in the United States, 1999–2023. The data demonstrates mortality trends categorized into three primary adult age groups: 25–44 years, 45–64 years, and ≥65 years

The age group ≥ 65 years had higher CMR (92.15), followed by the 45–64 year age group (19.82) and for the 25–44 year age group (1.18). For ≥ 65 year age group, the CMR increased drastically from 12.27 in 1999 to 114.39 in 2023 with an (AAPC: 11.68, 95% CI: 9.37 to 14.04; *p* < 0.01) with one joinpoint, this incline was notable between 1999 and 2005 (APC: 48.38, 95% CI: 36.11 to 61.76; *p* < 0.01) and between 2005 and 2023 (APC:1.58, 95% CI: 0.89 to 2.29; *p* = 0.01). 45–64 year age group also showed increasing mortality trend from 3.99 in 1999 to 27.25 in 2023 with an (AAPC: 8.22, 95% CI: 4.39 to 12.19; *p* = 0.001), with a changing trend showing three joinpoints, first the CMR increased from 3.99 in 1999 to 4.41 in 2001 (APC: 2.22, 95% CI: -30.01 to 49.31; *p* = 0.90), then it increased considerably from 4.41 in 2001 to 15.57 in 2005 (APC: 39.93, 95% CI: 22.73 to 59.52; *p* = 0.08), followed by a rise between 2005 and 2020 (APC: 4.22, 95% CI: 3.52 to 4.92; *p* < 0.01), then there was a gentler decline from 30.89 in 2020 to 27.25 in 2023 (APC: -3.64, 95% CI: -9.37 to 2.43; *p* = 0.21). For age group 25–44 year, the CMR increased from 0.28 in 1999 to 1.5 in 2023 (AAPC: 8.69, 95% CI: 6.77 to 10.64; *p* < 0.01) with two joinpoints identified, the rise was influential from 1999 to 2005 (APC: 29.80, 95% CI: 20.79 to 39.48; *p* < 0.01), and between 2005 and 2023 (APC: 2.45, 95% CI: 1.64 to 3.27; *p* = 0.03) (Supplemental Tables 2, 5; Fig. 5).

### Stratified by census region

**Fig. 6:**
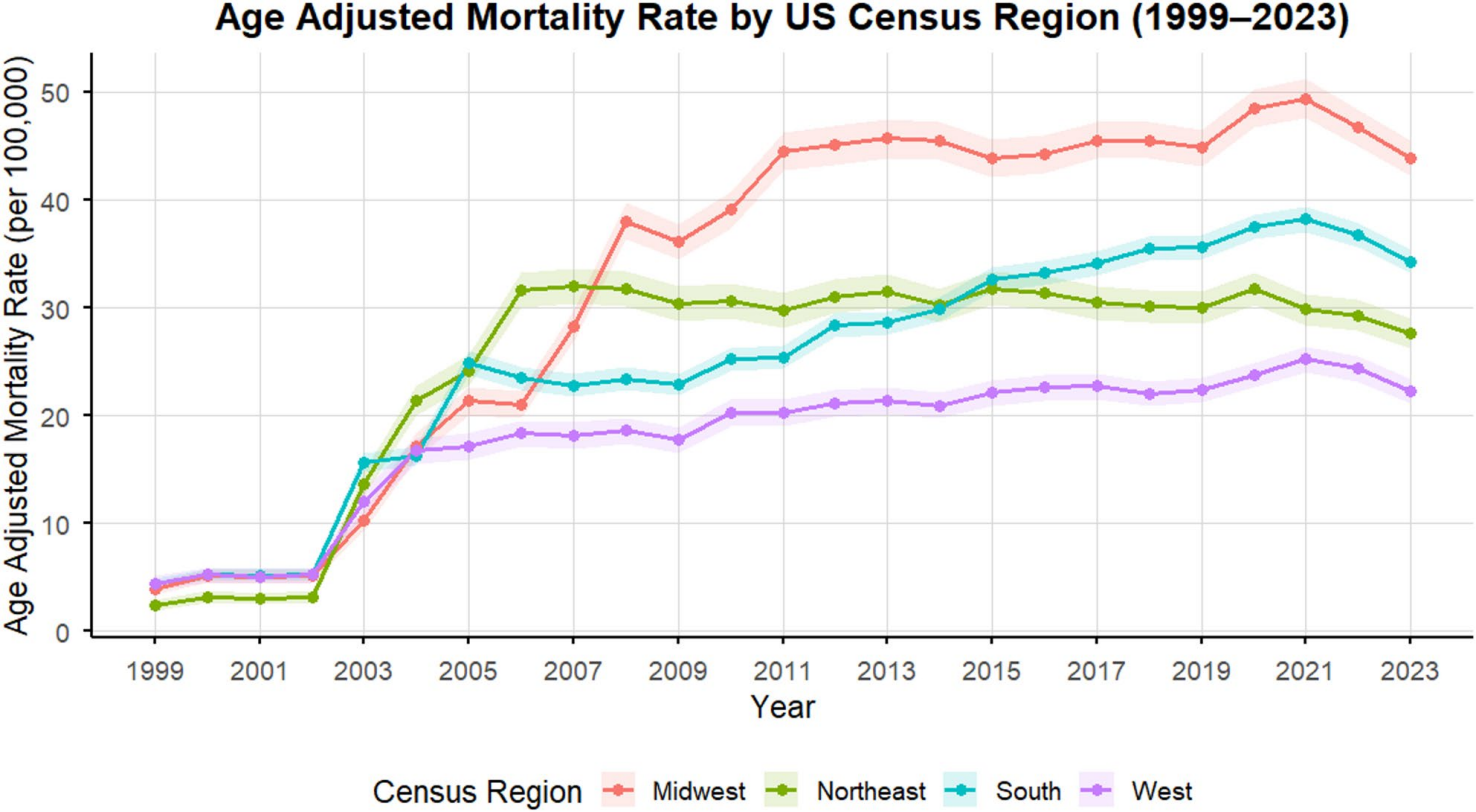
Ischemic Heart Disease Mortality with Documented Tobacco Use-Related Age-Adjusted Mortality Rates per 100,000, Stratified by Census Region, in Adults Aged ≥ 25 years in the United States, 1999–2023. The figure compares mortality trends across the four major United States census regions: Midwest, Northeast, South, and West

The highest AAMR was reported in the Midwest (32.96), followed by the South (25.01), the Northeast (24.91), and the West (18.01). In the Midwest region, the AAMR increased from 3.96 in 1999 to 43.87 in 2023 with an (AAPC: 11.34, 95% CI: 9,47 to 13.24; *p* < 0.01) with one joinpoint, this incline was significant between 1999 and 2008 increasing from 3.96 to 38.06 (APC: 30.49, 95% CI: 24.67 to 36.57; *p* < 0.01), and between 2008 and 2023 from 38.06 to 43.87 (APC: 1.23, 95% CI: 0.32 to 2.15; *p* = 0.01). For the South region, there was also a rise in AAMR from 4.4 in 1999 to 34.29 in 2023 with an (AAPC: 10.38, 95% CI: 7.98 to 12.83; *p* < 0.001) with one joinpoint identified, this rise was also noted between two stages, first from 4.4 in 1999 to 24.94 in 2005 (APC: 35.53, 95% CI: 23.84 to 48.33; *p* = 0.01), and then from 24.94 in 2005 34.29 in 2023 (APC: 3.08, 95% CI: 2.25 to 3.91; *p* < 0.01). The mortality for the Northeast region increased from 2.43 in 1999 to 27.68 in 2023 with an (AAPC: 12.54, 95% CI: 10.11 to 15.04; *p* < 0.001) with one joinpoint, first increasing notably from 2.43 in 1999 to 24.17 in 2005 (APC: 61.25, 95% CI: 47.28 to 76.56; *p* < 0.01), and then from 24.17 in 2005 to 27.68 in 2023 (APC: -0.16, 95% CI: -0.87 to 0.55; *p* = 0.63). For the West region, there was increase from 4.47 in 1999 to 22.25 in 2023 with an (AAPC: 7.09, 95% CI: 2.86 to 11.49; *p* = 0.09) with two joinpoints identified, this incline was exhibited from 4.47 in 1999 to 5.05 in 2001 (APC: -2.32, 95% CI: -29.72 to 35.75; *p* = 0.88), and then from 5.05 in 2001 to 16.76 in 2004 (APC: 56.71, 95% CI: 19.93 to 104.76; *p* = 0.02), followed by progressive increase from 16.76 in 2004 to 22.25 in 2023 (APC: 1.83, 95% CI: 1.41 to 2.24; *p* < 0.01) (Supplemental Tables 2, 6; Fig. 6).

### Mortality by states and place of death

The highest AAMR was observed from 1999 to 2020 in Vermont (AAMR: 68.81) and the lowest was observed in California (AAMR: 3.72). The states falling within the top 90th percentile included Arkansas, Delaware, North Dakota, Idaho, Kansas, Michigan, Montana, and South Dakota. States falling within the bottom percentile included Mississippi, Connecticut, and Alabama. From 2020 to 2023, the highest AAMR was observed in Vermont (AAMR: 73.59), and the lowest in California (AAMR: 3.23). States falling within the top 90th percentile included Arizona, Delaware, Idaho, Iowa, Kentucky, and Montana. States falling within the bottom percentile included Utah, Illinois, Florida, and Connecticut. State level estimates showed substantial variation, however, these findings should be interpreted cautiously given potential instability in states with small populations and possible coding variability. (Supplemental Tables 7, 8).

In a total of 1,497,919 deaths which occurred majority were recorded in descendants’ home (40.23%) followed by inpatients settings (27.89%), outpatient or emergency room settings (11.99%), nursing homes or long-term care settings (10.57%), hospice facilities (4.07%). A smaller proportion of deaths occurred in medical facilities - dead on arrival (0.85%), medical facilities - status unknown (0.008%), other locations (4.27%), and for 0.086% of cases, the place of death was unknown (Supplemental Table 9).

### Stratified by urbanization

**Fig. 7:**
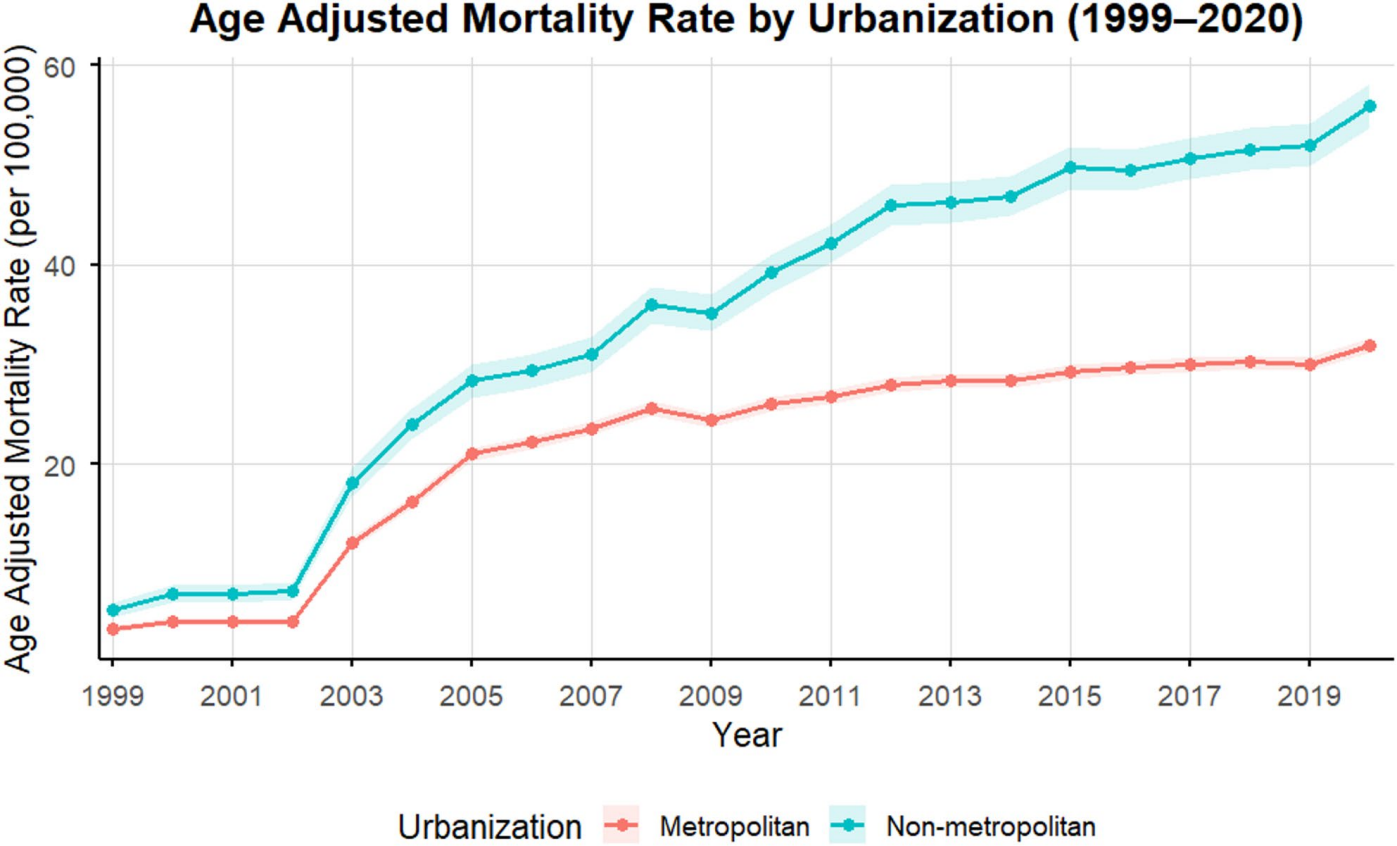
Ischemic Heart Disease Mortality with Documented Tobacco Use-Related Age-Adjusted Mortality Rates per 100,000, Stratified by Urban-Rural Status, in Adults Aged ≥ 25 years in the United States, 1999–2020. The line graph contrasts mortality trends between residents of metropolitan and non-metropolitan areas over the study period

Data on urbanization were available from the CDC only through 2020. Non-metropolitan areas reported a higher AAMR (35.73) with two joinpoints compared with metropolitan areas (22.95) with one joint point identified. In Non-metropolitan areas, the AAMR increased from 5.42 in 1999 to 55.88 in 2020 with an (AAPC: 12.91, 95% CI: 10.17 to 15.72; *p* < 0.01), there was a three phase incline, first increasing substantially from 5.42 in 1999 to 24.02 in 2004 (APC: 42.35, 95% CI: 29.04 to 57.05; *p* = 0.02), then the trend showed incline from 24.02 in 2004 to 45.94 in 2012 (APC: 7.94, 95% CI: 4.83 to 11.14; *p* = 0.07), followed by a moderate increase from 45.94 in 2012 to 55.88 in 2020 (APC: 2.19, 95% CI: 0.28 to 4.14; *p* = 0.03). For metropolitan areas, the AAMR increased from 3.56 in 1999 to 31.91 in 2020 with an (AAPC: 12.76, 95% CI: 10.39 to 15.19; *p* < 0.01), this incline was significant between 1999 and 2005 (APC: 43.70, 95% CI: 33.05 to 55.21; *p* < 0.01), and between 2005 and 2023 (APC: 2.34, 95% CI: 1.44 to 3.26; *p* = 0.04) (Supplemental Tables 2, 10; Fig. 7).

## Discussion

A steady rise in AAMRs due to IHD with documented tobacco use disorder was observed across the U.S. from 1999 to 2023, with a pronounced surge until 2015 and a subsequent plateau with slight increases thereafter. This trend contrasts with pre-existing literature reporting a global decline in deaths [13]. Mortality rates were consistently higher among males, NH White individuals, and adults aged ≥ 65 years. Regionally, the highest AAMRs were reported in non-metropolitan areas and the Midwest region, with the highest AAMR in Vermont, about 24 times that of California, which had the lowest AAMR.

IHD is one of the diseases with the highest mortality among CVDs. Over 9 million deaths from IHD have been reported annually [14]. IHD and tobacco use have a well-documented correlation. By promoting atherogenesis, atherosclerosis, and atherothrombosis through various mechanisms, including endothelial dysfunction, lipid oxidation, insulin resistance, prothrombotic and proinflammatory effects, tobacco use is a major and modifiable risk factor that contributes to the onset and progression of IHD [15]. Even though smoking prevalence has declined over the past years [16], the improvements in documentation may contribute to the early upward trend. Although the marked increase observed during the early study years may partially reflect evolving documentation practices for ICD-10 code F17 on death certificates, sensitivity analysis restricted to the post-2005 period demonstrated persistence of the increasing mortality trend, suggesting that the findings cannot be fully attributed to coding changes alone. Nevertheless, temporal variability in tobacco use disorder documentation remains an important limitation when interpreting long-term mortality trends. Additionally, the shift from relatively limited ICD-9 codes to the ICD-10 codes may also contribute to these findings. While ICD-9 offered only two codes for tobacco use, ICD-10 introduced over twenty codes under the category “F17”, which encompasses a broad range of tobacco use. An increase in the documentation of all kinds of tobacco use may also justify the observed rise in mortality despite the evident decrease in tobacco use.

Pre-existing literature largely reports declining prevalence and mortality trends for both IHD and tobacco use worldwide [13, 16], often using smoking-attributable mortality models or population-based epidemiologic estimates. In contrast, our analysis examined death certificate data among decedents with documented tobacco use disorder, representing a distinct endpoint that is influenced not only by disease burden but also by documentation and coding practices over time. Improvements in recognition and reporting of tobacco use disorder on death certificates may therefore partly contribute to the observed rise. Furthermore, over 71% of deaths occurred in individuals aged ≥ 65 years, a population with historically higher cumulative smoking exposure and a slower decline in smoking prevalence [16]. In addition, reductions in mortality may lag behind decreases in smoking prevalence because of the prolonged latent period between tobacco exposure and IHD development [17]. Persistent residual cardiovascular risk after smoking cessation may also contribute to continued mortality burden [18]. Lifestyle modification, industrialization, and advancements have led to a busy life and increased stress levels, coupled with sedentary habits. Similarly, the prevalence of comorbid conditions, including obesity, diabetes, hypertension, and dyslipidemia, has increased significantly over the years [19]. All these serve as modifiable risk factors for IHD.

The COVID-19 pandemic serves as a major confounding factor for mortality data 2020 onwards, as it contributes to cardiovascular complications and exacerbates the disparities in healthcare [20]. The recent introduction and increased use of high-sensitivity troponin has led to increased sensitivity in the diagnosis of IHD, which improves myocardial injury detection [21]. As a result, deaths previously labelled “unspecified” or attributed to alternative causes can now be classified as IHD on death certificates. This could give rise to an apparent increase in recorded IHD deaths rather than a true increase in underlying mortality.

When stratified by sex, discrepancy was evident with males having higher AAMRs. This disparity may be attributed to the male gender itself being a risk factor for IHD [22]. The difference is further influenced by a greater susceptibility of men to associated risk factors. Substance abuse, including alcohol and smoking, and stress levels are higher in men as compared to women [23], and play a pivotal role in acquiring the disease. Men accumulate more visceral fat as compared to women, in whom fat accumulates mainly in a subcutaneous fashion, which is metabolically less harmful [24]. On the other hand, women are at a lower risk due to certain physiological factors; estrogen in women, specifically via its vasodilatory, anti-inflammatory, and thrombogenic effects [25], exerts a protective role. Moreover, estrogen also lowers LDL and increases HDL [26]. 

Disparities in deaths were evident across various racial groups in the U.S. as well. Mortality was the highest in NH White individuals, while it was the lowest in Asians. The higher death rates of NH Whites possibly emerge from their proposed favourable genetic makeup [27], though more research is needed in this aspect. The preponderance of sedentary lifestyles and dietary habits of NH Whites further contribute to increased mortality. Additionally, better access to healthcare facilities compared to the other races leads to a greater number of diagnosed and documented cases.

Age is a risk factor for IHD, and adults aged ≥ 65 years exhibit the highest mortality due to a cumulative burden posed by several factors. Vascular damage over time [28], longer exposure to risk factors, decline in protective mechanisms [29], and increased comorbidities [30] are major contributors.

Geographically, the Midwest had the highest AAMR, with Vermont exhibiting the highest state-level AAMR. These geographical trends may reveal disparities in healthcare infrastructure, socioeconomic status, and health-seeking behavior of the general population. Vermont is among the least populous states in the U.S., and is more susceptible to instability in AAMRs due to small figures. Even minor changes in the number of reported cases can lead to substantial fluctuations in mortality rate over time. In contrast, lowest AAMRs in California may be associated with its relatively aggressive tobacco control [31] and comparatively lower prevalence of smoking among the states of the U.S., as supported by data from American Lung Association 2025 report [32]. Variations in coding practices across the states might also contribute to the observed differences.

Non-metropolitan (rural) areas persistently exhibited higher AAMRs than metropolitan (urban) areas. Several factors serve as a barrier to optimal healthcare in rural settings, such as lack of resources, education, and awareness, scarcity of specialists as well as primary care physicians, coupled with physician burnout, and fewer healthcare facilities [33, 34]. Lack of access to screening programs, socioeconomic challenges, and insurance problems may also contribute. A greater percentage of men inhabiting the rural areas can also justify this discrepancy [35]. 

Our study utilizes the standard U.S. healthcare data spanning over 25 years, permitting a robust analysis. To our knowledge this is among the first studies that analyze and stratify the mortality burden where both IHD and tobacco use are listed on the death certificates. While the CDC WONDER database offers a nationally representative dataset for analyzing mortality trends, certain limitations need to be acknowledged. The CDC WONDER ICD-10 system used for classifying mortalities could present with misdiagnosis, underreporting or omission of either IHD or tobacco as a contributor to death, particularly more for tobacco use which has been historically undercoded. This might lead to misclassification or underestimation of mortality burden. Additionally, it fails to account for detailed patient-level information such as frequency and duration of smoking, cumulative exposure (pack years), comorbidities, medication use, diagnostic criteria, and socioeconomic variables, which could provide a deeper understanding of IHD and tobacco outcomes. Also, observed trends may be influenced by changes in death certification practices, coding behavior, and reporting completeness over time. Individuals with significant prior smoking history who recently quit are likely to be misclassified despite harboring elevated risk, underestimating the true association between IHD and tobacco use. The place of death data reported in our study were derived from death certificates and analyzed descriptively without assessment of emergency medical services access, autopsy findings, or sudden cardiac death classifications. This may reflect differences in access or certification. That’s why it should be interpreted with caution. Furthermore, an important limitation of this study is the reliance on ICD-10 code F17 as a contributing cause of death, which reflects documented tobacco use disorder on death certificates rather than a direct measure of smoking-attributable mortality. Documentation of F17 may vary across regions, certifiers, and study years, and part of the observed increase may reflect evolving coding practices over time. For urbanization, data from 1999 to 2020 was utilized as per its availability, limiting the applicability of our findings to more recent years. Lastly, the use of 2000 standard U.S. population for age adjustment may not fully account for the changes that occurred over the past 25 years.

## Conclusion

A persistent overall increase in IHD fatalities with tobacco use as a contributing factor was observed, with evident disparities across various demographic and geographic subgroups. This calls for targeted interventions and research focused especially on the high-risk population.

## Supplementary Information

Supplementary Material 1.

## Data Availability

The data that support the findings of this study are openly available in CDC‐WONDER at [https://wonder.cdc.gov/]. The data supporting the findings of this study were obtained from the CDC WONDER online database (Centers for Disease Control and Prevention Wide‐ranging Online Data for Epidemiologic Research). Further inquiries can be directed to the corresponding author.

## Abbreviations

IHD: Ischemic Heart Disease
CVDs: Cardiovascular Diseases
AAMR: Age-Adjusted Mortality Rate
AAPC: Average Annual Percent Change
APC: Annual Percent Change
CDC: Centers for Disease Control and Prevention
CI: Confidence Interval
CMR: Crude Mortality Rate
COVID-19: Coronavirus Disease 2019
DALY: Disability-adjusted Life-Years
HDL: High-density Lipoprotein
ICD-10: International Statistical Classification of Diseases and Related Health Problems, 10th Revision
LDL: Low-density Lipoprotein
NH: Non-Hispanic
STROBE: Strengthening the Reporting of Observational Studies in Epidemiology
WONDER: Wide-Ranging Online Data for Epidemiologic Research
