# Supplementary material for: Ischemic heart disease mortality with documented tobacco use in the United States, 1999–2023: a nationwide population-based analysis
Source: Arch Public Health. 2026 Jul 18;84:164. doi: 10.1186/s13690-026-02013-y (PMC13383482; doi:10.1186/s13690-026-02013-y)
Supplement: Supplementary file 1 — Supplementary Material 1. [file 13690_2026_2013_MOESM1_ESM.docx]

**Supplementary Tables:**

**Supplemental Table 1:** Ischemic heart disease mortality with documented tobacco use per 100,000 adults aged ≥ 25 years in the United States, stratified by sex and race, 1999–2023.

| Year | Overall | Female | Male | NH White | NH Black or African American | NH Asian or Pacific Islander | NH American Indian or Alaska Native | Hispanic or Latino |
| --- | --- | --- | --- | --- | --- | --- | --- | --- |
| 1999 | 6,918 | 2,296 | 4,622 | 6,322 | 517 | 36 | 43 | 172 |
| 2000 | 8,633 | 2,953 | 5,680 | 7,937 | 576 | 52 | 68 | 194 |
| 2001 | 8,598 | 2,893 | 5,705 | 7,890 | 570 | 58 | 80 | 180 |
| 2002 | 8,990 | 3,115 | 5,875 | 8,272 | 581 | 57 | 80 | 200 |
| 2003 | 24,979 | 8,256 | 16,723 | 23,032 | 1,638 | 141 | 168 | 968 |
| 2004 | 33,819 | 11,296 | 22,523 | 31,170 | 2,178 | 183 | 288 | 1,052 |
| 2005 | 43,648 | 14,196 | 29,452 | 40,105 | 2,953 | 251 | 339 | 1,383 |
| 2006 | 46,730 | 15,154 | 31,576 | 42,799 | 3,187 | 350 | 394 | 1,571 |
| 2007 | 50,661 | 16,281 | 34,380 | 46,455 | 3,392 | 425 | 389 | 1,567 |
| 2008 | 56,796 | 18,242 | 38,554 | 52,005 | 3,940 | 458 | 393 | 1,660 |
| 2009 | 55,613 | 17,698 | 37,915 | 50,734 | 3,979 | 493 | 407 | 1,706 |
| 2010 | 60,978 | 19,321 | 41,657 | 55,662 | 4,331 | 536 | 449 | 1,799 |
| 2011 | 64,999 | 20,240 | 44,759 | 59,398 | 4,619 | 519 | 463 | 1,980 |
| 2012 | 70,322 | 21,852 | 48,470 | 64,060 | 5,158 | 591 | 513 | 2,155 |
| 2013 | 72,871 | 22,485 | 50,386 | 66,206 | 5,482 | 648 | 535 | 2,339 |
| 2014 | 74,895 | 22,889 | 52,006 | 67,748 | 5,870 | 664 | 613 | 2,492 |
| 2015 | 79,498 | 24,245 | 55,253 | 71,653 | 6,442 | 722 | 681 | 2,871 |
| 2016 | 82,284 | 25,077 | 57,207 | 73,744 | 7,101 | 776 | 663 | 2,799 |
| 2017 | 85,482 | 25,850 | 59,632 | 76,494 | 7,371 | 857 | 760 | 2,925 |
| 2018 | 88,110 | 26,603 | 61,507 | 78,865 | 7,615 | 894 | 736 | 3,168 |
| 2019 | 89,828 | 27,052 | 62,776 | 80,202 | 7,985 | 946 | 695 | 3,316 |
| 2020 | 97,259 | 29,128 | 68,131 | 86,000 | 9,270 | 1,090 | 899 | 3,935 |
| 2021 | 97,545 | 29,428 | 68,117 | 86,355 | 9,006 | 864 | 764 | 3,858 |
| 2022 | 96,900 | 29,543 | 67,357 | 85,679 | 8,987 | 913 | 779 | 3,834 |
| 2023 | 91,563 | 28,069 | 63,494 | 80,584 | 8,833 | 874 | 698 | 3,757 |

NH, non-Hispanic

**Supplemental Table 2:** Annual percent change (APC) in age-adjusted mortality rates (AAMRs) for ischemic heart disease mortality with documented tobacco use per 100,000 adults aged ≥25 years in the United States, 1999–2023.

| Year Interval | APC (95% CI) |
| --- | --- |
| Overall | |
| 1999-2005 | 40.09 (29.38 to 51.68) |
| 2005-2013 | 4.25 (1.10 to 7.51) |
| 2013-2023 | 0.87 (−0.61 to 2.38) |
| Sex | |
| Women | |
| 1999-2005 | 41.88 (31.51 to 53.06) |
| 2005-2023 | 2.00 (1.32 to 2.69) |
| Men | |
| 1999-2005 | 41.33 (30.36 to 53.23) |
| 2005-2015 | 3.61 (1.51 to 5.75) |
| 2015-2023 | 0.19 (−1.79 to 2.20) |
| Race | |
| NH American Indian or Alaska Native | |
| 1999-2005 | 38.74 (24.78 to 54.25) |
| 2005-2023 | 0.52 (−0.35 to 1.40) |
| NH Asian or Pacific Islander | |
| 1999-2006 | 36.98 (29.86 to 44.49) |
| 2006-2020 | 1.62 (0.84 to 2.40) |
| 2020-2023 | −6.59 (−12.22 to −0.60) |
| NH Black or African American | |
| 1999-20005 | 39.60 (28.81 to 51.29) |
| 2005-2020 | 4.19 (3.18 to 5.21) |
| 2020-2023 | **−**1.71 (−9.23 to 6.44) |
| NH White | |
| 1999-2005 | 39.45 (29.28 to 50.41) |
| 2005-2013 | 4.37 (1.42 to 7.42) |
| 2013-2023 | 0.93 (−0.46 to 2.35) |
| Hispanic or Latino | |
| 1999-2004 | 55.65 (32.53 to 82.80) |
| 2004-2023 | 1.30 (0.47 to 2.14) |
| Census Region | |
| Northeast | |
| 1999-2005 | 61.26 (47.28 to 76.56) |
| 2005-2023 | −0.17 (−0.88 to 0.55) |
| Midwest | |
| 1999-2008 | 30.49 (24.68 to 36.57) |
| 2008-2023 | 1.23 (0.32 to 2.15) |
| South | |
| 1999-2005 | 35.54 (23.84 to 48.33) |
| 2005-2023 | 3.08 (2.26 to 3.91) |
| West | |
| 1999-2001 | −2.32 (−29.72 to 35.76) |
| 2001-2004 | 56.71 (19.94 to 104.77) |
| 2004-2023 | 1.82 (1.41 to 2.24) |
| Urban-rural classification | |
| Metropolitan | |
| 1999-2005 | 43.71 (33.05 to 55.21) |
| 2005-2020 | 2.35 (1.44 to 3.26) |
| Non-Metropolitan | |
| 1999-2004 | 42.36 (29.04 to 57.05) |
| 2004-2012 | 7.94 (4.83 to 11.14) |
| 2012-2023 | 2.20 (0.29 to 4.14) |
| Age Groups | |
| Age 25-44 years | |
| 1999-2005 | 29.80 (20.79 to 39.48) |
| 2005-2023 | 2.45 (1.64 to 3.27) |
| Age 45-64 years | |
| 1999-2001 | 2.23 (−30.01 to 49.32) |
| 2001-2005 | 39.92 (22.73 to 59.53) |
| 2005-2020 | 4.22 (3.53 to 4.93) |
| 2020-2023 | −3.65 (−9.37 to 2.44) |
| Age 65+ years | |
| 1999-2005 | 48.38 (36.11 to 61.76) |
| 2005-2023 | 1.59 (0.89 to 2.29) |

CI, Confidence Interval; NH, non-Hispanic

**Supplemental Table 3:** Overall and sex-stratified age-adjusted mortality rates (AAMRs) for ischemic heart disease mortality with documented tobacco use per 100,000 adults aged ≥ 25 years in the United States, 1999–2023.

| Year | Overall | Female | Male |
| --- | --- | --- | --- |
| 1999 | 3.9 (3.81-3.99) | 2.29 (2.19-2.38) | 6.02 (5.84-6.19) |
| 2000 | 4.81 (4.71-4.91) | 2.89 (2.79-3) | 7.27 (7.07-7.46) |
| 2001 | 4.7 (4.6-4.8) | 2.8 (2.7-2.9) | 7.14 (6.95-7.33) |
| 2002 | 4.81 (4.71-4.91) | 2.95 (2.84-3.05) | 7.21 (7.02-7.39) |
| 2003 | 13.22 (13.05-13.38) | 7.63 (7.46-7.79) | 20.89 (20.57-21.21) |
| 2004 | 17.66 (17.47-17.85) | 10.28 (10.09-10.47) | 27.74 (27.37-28.1) |
| 2005 | 22.34 (22.13-22.55) | 12.71 (12.5-12.92) | 35.59 (35.18-36) |
| 2006 | 23.47 (23.25-23.68) | 13.34 (13.12-13.55) | 37.3 (36.88-37.72) |
| 2007 | 24.92 (24.7-25.14) | 14.12 (13.9-14.34) | 39.57 (39.14-40) |
| 2008 | 27.42 (27.2-27.65) | 15.49 (15.27-15.72) | 43.43 (42.99-43.87) |
| 2009 | 26.28 (26.06-26.5) | 14.79 (14.57-15.01) | 41.67 (41.25-42.1) |
| 2010 | 28.38 (28.15-28.6) | 15.96 (15.47-16.19) | 44.88 (44.44-45.32) |
| 2011 | 29.43 (29.2-29.65) | 16.36 (16.13-16.59) | 46.69 (46.25-47.13) |
| 2012 | 31.05 (30.82-31.28) | 17.28 (17.05-17.52) | 49.05 (48.6-49.5) |
| 2013 | 31.38 (31.15-31.61) | 17.4 (17.17-17.63) | 49.58 (49.14-50.02) |
| 2014 | 31.43 (31.21-31.66) | 17.31 (17.09-17.54) | 49.7 (49.27-50.14) |
| 2015 | 32.6 (32.37-32.83) | 17.93 (17.7-18.16) | 51.54 (51.1-51.98) |
| 2016 | 32.97 (32.47-33.2) | 18.13 (17.9-18.36) | 51.87 (51.43-52.3) |
| 2017 | 33.4 (33.17-33.63) | 18.25 (18.03-18.48) | 52.71 (52.28-53.14) |
| 2018 | 33.67 (33.44-33.89) | 18.37 (18.14-18.59) | 53.08 (52.65-53.51) |
| 2019 | 33.61 (33.39-33.84) | 18.3 (18.08-18.52) | 52.9 (52.48-53.33) |
| 2020 | 35.7 (35.47-35.93) | 19.43 (19.2-19.66) | 56.17 (55.74-56.6) |
| 2021 | 36.12 (35.88-36.35) | 19.94 (19.71-20.17) | 56.19 (55.75-56.62) |
| 2022 | 34.7 (34.48-34.92) | 19.3 (19.08-19.53) | 54.04 (53.62-54.46) |
| 2023 | 32.37 (32.16-32.59) | 18.13 (17.92-18.35) | 49.91 (49.51-50.3) |

**Supplemental Table 4:** Age-adjusted mortality rates (AAMRs) for ischemic heart disease mortality with documented tobacco use per 100,000 adults aged ≥ 25 years, stratified by race, in the United States, 1999–2023.

| Year | NH White | NH Black or African American | NH Asian or Pacific Islander | NH American Indian or Alaska Native | Hispanic or Latino |
| --- | --- | --- | --- | --- | --- |
| 1999 | 4.06 (3.96-4.16) | 3.16 (2.88-3.44) | 0.74 (0.51-1.04) | 4.55 (3.2-6.27) | 1.79 (1.51-2.08) |
| 2000 | 5.09 (4.97-5.2) | 3.44 (3.16-3.72) | 1.04 (0.76-1.39) | 6.81 (5.19-8.76) | 1.91 (1.63-2.2) |
| 2001 | 4.94 (4.83-5.04) | 3.36 (3.08-3.64) | 1.06 (0.79-1.39) | 7.15 (5.58-9.04) | 1.74 (1.47-2) |
| 2002 | 5.09 (4.98-5.2) | 3.3 (3.03-3.58) | 1.03 (0.77-1.36) | 7.02 (5.47-8.87) | 1.78 (1.52-2.04) |
| 2003 | 13.97 (13.79-14.15) | 9.46 (9-9.93) | 2.44 (2.02-2.86) | 14.26 (11.96-16.55) | 8.72 (8.14-9.29) |
| 2004 | 18.66 (18.46-18.87) | 12.31 (11.78-12.83) | 3.04 (2.58-3.5) | 25.09 (21.97-28.21) | 9.03 (8.46-9.6) |
| 2005 | 23.61 (23.38-23.84) | 16.22 (15.62-16.82) | 3.82 (3.33-4.31) | 27.49 (24.33-30.65) | 11.03 (10.42-11.63) |
| 2006 | 24.79 (24.56-25.03) | 16.85 (16.25-17.45) | 5.19 (4.62-5.75) | 30.5 (27.25-33.75) | 11.92 (11.31-12.54) |
| 2007 | 26.41 (26.17-26.66) | 17.64 (17.03-18.25) | 5.83 (5.25-6.4) | 29.18 (26.03-32.32) | 11.33 (10.74-11.91) |
| 2008 | 29.06 (28.81-29.31) | 19.97 (19.33-20.61) | 6.25 (5.66-6.84) | 27.6 (24.65-30.55) | 11.42 (10.85-11.99) |
| 2009 | 27.85 (27.61-28.09) | 19.47 (18.84-20.09) | 6.08 (5.52-6.63) | 27.29 (24.42-30.16) | 10.98 (10.44-11.53) |
| 2010 | 30.16 (29.9-30.41) | 20.8 (20.16-21.44) | 6.6 (6.02-7.17) | 29.19 (26.27-32.1) | 11.34 (10.79-11.89) |
| 2011 | 31.44 (31.18-31.7) | 21.09 (20.46-21.72) | 5.8 (5.29-6.32) | 26.95 (24.3-29.29) | 11.45 (10.93-11.98) |
| 2012 | 33.16 (32.91-33.42) | 22.89 (22.24-23.53) | 6.35 (5.83-6.88) | 28.94 (26.25-31.62) | 11.94 (11.42-12.46) |
| 2013 | 33.53 (33.27-33.79) | 23.25 (22.61-23.89) | 6.41 (5.9-6.91) | 28.36 (25.79-30.94) | 12.33 (11.81-12.85) |
| 2014 | 33.54 (33.28-33.8) | 24.24 (23.6-24.88) | 6.3 (5.81-6.79) | 31.72 (29.05-34.39) | 12.35 (11.85-12.86) |
| 2015 | 34.82 (34.56-35.07) | 25.59 (24.95-26.24) | 6.27 (5.81-6.74) | 33.2 (30.55-35.85) | 13.45 (12.94-13.96) |
| 2016 | 35.08 (34.83-35.34) | 27.18 (26.53-27.83) | 6.5 (6.03-6.96) | 31.5 (28.96-34.03) | 12.47 (11.99-12.95) |
| 2017 | 35.6 (35.35-35.86) | 27.27 (26.63-27.91) | 6.81 (6.34-7.27) | 33.8 (31.26-36.34) | 12.36 (11.89-12.82) |
| 2018 | 36 (35.74-36.25) | 27.43 (26.8-28.07) | 6.76 (6.31-7.21) | 31.55 (29.16-33.94) | 12.95 (12.48-13.41) |
| 2019 | 35.91 (35.66-36.16) | 27.93 (27.3-28.56) | 6.84 (6.4-7.28) | 28.23 (26.03-30.42) | 12.95 (12.49-13.4) |
| 2020 | 37.95 (37.69-38.21) | 31.52 (30.86-32.18) | 7.45 (7-7.9) | 35.15 (32.75-37.55) | 14.65 (14.18-15.13) |
| 2021 | 38.86 (38.6-39.12) | 30.72 (30.06-31.38) | 6.34 (5.91-6.77) | 32.18 (29.8-34.56) | 14.05 (13.59-14.51) |
| 2022 | 37.31 (37.06-37.57) | 30.25 (29.6-30.89) | 6.33 (5.91-6.74) | 31.21 (28.95-33.47) | 13.37 (12.93-13.81) |
| 2023 | 34.77 (34.53-35.02) | 28.91 (28.29-29.53) | 5.75 (5.37-6.14) | 26.9 (24.85-28.95) | 12.37 (11.96-12.78) |

NH, non-Hispanic

**Supplemental Table 5:** Crude mortality rates (CMRs) for ischemic heart disease mortality with documented tobacco use per 100,000 adults, stratified by age groups, in the United States, 1999–2023.

| **Year** | **Age 25-44 Years** | **Age 45-64 Years** | **Age 65+ Years** |
| --- | --- | --- | --- |
| 1999 | 0.28 (0.24-0.31) | 3.99 (3.84-4.15) | 12.27 (11.91-12.64) |
| 2000 | 0.36 (0.32-0.4) | 4.61 (4.44-4.78) | 15.63 (15.21-16.04) |
| 2001 | 0.33 (0.29-0.37) | 4.41 (4.24-4.57) | 15.51 (15.1-15.93) |
| 2002 | 0.36 (0.32-0.4) | 4.6 (4.44-4.76) | 15.83 (15.42-16.25) |
| 2003 | 0.7 (0.64-0.76) | 9.76 (9.53-10) | 49.28 (48.55-50.01) |
| 2004 | 0.86 (0.8-0.92) | 12.43 (12.17-12.69) | 67.08 (66.24-67.92) |
| 2005 | 1.09 (1.02-1.16) | 15.57 (15.29-15.86) | 85.57 (84.62-86.51) |
| 2006 | 1.17 (1.1-1.25) | 16.35 (16.07-16.64) | 90.03 (89.07-91) |
| 2007 | 1.13 (1.06-1.2) | 17.93 (17.64-18.23) | 94.92 (93.94-95.91) |
| 2008 | 1.2 (1.12-1.27) | 19.23 (18.92-19.53) | 104.94 (103.92-105.96) |
| 2009 | 1.18 (1.11-1.25) | 18.78 (18.48-19.08) | 99.86 (98.87-100.84) |
| 2010 | 1.28 (1.2-1.35) | 20.47 (20.16-20.78) | 107.4 (106.39-108.41) |
| 2011 | 1.36 (1.28-1.43) | 22.03 (21.71-22.35) | 110.28 (109.27-111.29) |
| 2012 | 1.46 (1.38-1.54) | 23.46 (23.13-23.79) | 115.13 (114.12-116.15) |
| 2013 | 1.43 (1.35-1.51) | 24.19 (23.86-24.53) | 115.38 (114.38-116.37) |
| 2014 | 1.36 (1.28-1.44) | 24.55 (24.22-24.89) | 115.13 (114.15-116.1) |
| 2015 | 1.44 (1.36-1.53) | 25.61 (25.27-25.95) | 118.81 (117.83-119.79) |
| 2016 | 1.41 (1.33-1.49) | 26.61 (26.26-26.96) | 119.13 (118.17-120.1) |
| 2017 | 1.4 (1.32-1.48) | 27.3 (26.95-27.66) | 120.4 (119.45-121.36) |
| 2018 | 1.41 (1.33-1.49) | 27.84 (27.48-28.19) | 121.16 (120.22-122.1) |
| 2019 | 1.51 (1.43-1.59) | 27.99 (27.63-28.35) | 120.58 (119.65-121.5) |
| 2020 | 1.77 (1.68-1.85) | 30.89 (30.51-31.27) | 126.01 (125.08-126.94) |
| 2021 | 1.84 (1.75-1.93) | 30.69 (30.31-31.06) | 125.85 (124.92-126.78) |
| 2022 | 1.73 (1.64-1.82) | 29.13 (28.76-29.5) | 123.4 (122.5-124.31) |
| 2023 | 1.5 (1.42-1.58) | 27.25 (26.89-27.61) | 114.39 (113.53-115.25) |

**Supplemental Table 6:** Age-adjusted mortality rates (AAMRs) for ischemic heart disease mortality with documented tobacco use per 100,000 adults aged ≥ 25 years, stratified by census regions, in the United States, 1999–2023.

| **Census Region** | **Year** | **Age Adjusted Rate (95% CI)** |
| --- | --- | --- |
| Northeast | 1999 | 2.43 (2.27-2.59) |
| Northeast | 2000 | 3.23 (3.05-3.42) |
| Northeast | 2001 | 3.04 (2.86-3.21) |
| Northeast | 2002 | 3.22 (3.04-3.4) |
| Northeast | 2003 | 13.66 (13.29-14.03) |
| Northeast | 2004 | 21.44 (20.98-21.9) |
| Northeast | 2005 | 24.17 (23.69-24.66) |
| Northeast | 2006 | 31.62 (31.07-32.18) |
| Northeast | 2007 | 32.03 (31.47-32.58) |
| Northeast | 2008 | 31.74 (31.19-32.29) |
| Northeast | 2009 | 30.38 (29.85-30.92) |
| Northeast | 2010 | 30.65 (30.12-31.19) |
| Northeast | 2011 | 29.79 (29.26-30.31) |
| Northeast | 2012 | 31.06 (30.53-31.59) |
| Northeast | 2013 | 31.56 (31.03-32.1) |
| Northeast | 2014 | 30.23 (29.71-30.74) |
| Northeast | 2015 | 31.81 (31.28-32.33) |
| Northeast | 2016 | 31.44 (30.92-31.96) |
| Northeast | 2017 | 30.48 (29.97-30.98) |
| Northeast | 2018 | 30.15 (29.66-30.65) |
| Northeast | 2019 | 30 (29.51-30.5) |
| Northeast | 2020 | 31.79 (31.29-32.3) |
| Northeast | 2021 | 29.86 (29.37-30.35) |
| Northeast | 2022 | 29.31 (28.83-29.78) |
| Northeast | 2023 | 27.68 (27.22-28.14) |
| Midwest | 1999 | 3.96 (3.76-4.15) |
| Midwest | 2000 | 5.16 (4.94-5.38) |
| Midwest | 2001 | 5.07 (4.86-5.29) |
| Midwest | 2002 | 5.18 (4.96-5.39) |
| Midwest | 2003 | 10.23 (9.93-10.53) |
| Midwest | 2004 | 17.18 (16.79-17.57) |
| Midwest | 2005 | 21.37 (20.94-21.8) |
| Midwest | 2006 | 21.03 (20.6-21.45) |
| Midwest | 2007 | 28.32 (27.83-28.81) |
| Midwest | 2008 | 38.06 (37.5-38.62) |
| Midwest | 2009 | 36.15 (35.6-36.69) |
| Midwest | 2010 | 39.09 (38.53-39.66) |
| Midwest | 2011 | 44.48 (43.89-45.08) |
| Midwest | 2012 | 45.09 (44.49-45.68) |
| Midwest | 2013 | 45.7 (45.11-46.3) |
| Midwest | 2014 | 45.47 (44.88-46.05) |
| Midwest | 2015 | 43.88 (43.3-44.45) |
| Midwest | 2016 | 44.26 (43.69-44.83) |
| Midwest | 2017 | 45.53 (44.69-46.11) |
| Midwest | 2018 | 45.56 (44.99-46.13) |
| Midwest | 2019 | 44.87 (44.31-45.43) |
| Midwest | 2020 | 48.51 (47.93-49.08) |
| Midwest | 2021 | 49.42 (48.83-50.01) |
| Midwest | 2022 | 46.73 (46.17-47.29) |
| Midwest | 2023 | 43.87 (43.33-44.41) |
| South | 1999 | 4.4 (4.24-4.56) |
| South | 2000 | 5.26 (5.09-5.44) |
| South | 2001 | 5.23 (5.05-5.4) |
| South | 2002 | 5.28 (5.1-5.45) |
| South | 2003 | 15.63 (15.33-15.92) |
| South | 2004 | 16.26 (15.96-16.56) |
| South | 2005 | 24.94 (24.57-25.32) |
| South | 2006 | 23.48 (23.13-23.84) |
| South | 2007 | 22.82 (22.48-23.17) |
| South | 2008 | 23.45 (23.1-23.79) |
| South | 2009 | 22.93 (22.59-23.27) |
| South | 2010 | 25.23 (24.88-25.58) |
| South | 2011 | 25.43 (25.08-25.78) |
| South | 2012 | 28.36 (28-28.73) |
| South | 2013 | 28.61 (28.24-28.97) |
| South | 2014 | 29.94 (29.57-30.3) |
| South | 2015 | 32.63 (32.25-33) |
| South | 2016 | 33.31 (32.93-33.68) |
| South | 2017 | 34.16 (33.79-34.54) |
| South | 2018 | 35.5 (35.13-35.88) |
| South | 2019 | 35.6 (35.23-35.97) |
| South | 2020 | 37.55 (37.17-37.93) |
| South | 2021 | 38.22 (37.83-38.6) |
| South | 2022 | 36.79 (36.42-37.16) |
| South | 2023 | 34.29 (33.94-34.64) |
| West | 1999 | 4.47 (4.25-4.69) |
| West | 2000 | 5.24 (5-5.47) |
| West | 2001 | 5.05 (4.82-5.28) |
| West | 2002 | 5.26 (5.03-5.49) |
| West | 2003 | 11.99 (11.65-12.33) |
| West | 2004 | 16.76 (16.35-17.16) |
| West | 2005 | 17.16 (16.75-17.56) |
| West | 2006 | 18.35 (17.94-18.76) |
| West | 2007 | 18.14 (17.73-18.55) |
| West | 2008 | 18.6 (18.19-19) |
| West | 2009 | 17.75 (17.36-18.14) |
| West | 2010 | 20.34 (19.92-20.75) |
| West | 2011 | 20.25 (19.85-20.66) |
| West | 2012 | 21.17 (20.76-21.59) |
| West | 2013 | 21.38 (20.98-21.79) |
| West | 2014 | 20.91 (20.52-21.31) |
| West | 2015 | 22.09 (21.69-22.49) |
| West | 2016 | 22.6 (22.2-23) |
| West | 2017 | 22.74 (22.34-23.14) |
| West | 2018 | 22.06 (21.67-22.44) |
| West | 2019 | 22.43 (22.05-22.81) |
| West | 2020 | 23.76 (23.37-24.15) |
| West | 2021 | 25.24 (24.83-25.64) |
| West | 2022 | 24.37 (23.98-24.76) |
| West | 2023 | 22.25 (21.88-22.62) |

CI, Confidence Interval

**Supplemental Table 7:** Age-adjusted mortality rates (AAMRs) for ischemic heart disease mortality with documented tobacco use per 100,000 adults aged ≥ 25 years, stratified by state, in the United States, 1999–2020.

| **State** | **Age Adjusted Rate (95% CI)** |
| --- | --- |
| Alabama | 8.59 (8.39-8.8) |
| Alaska | 19.94 (18.82-21.06) |
| Arizona | 22.51 (22.21-22.8) |
| Arkansas | 27.42 (26.95-27.89) |
| California | 3.72 (3.66-3.77) |
| Colorado | 24.16 (23.79-24.53) |
| Connecticut | 18.7 (18.36-19.05) |
| Delaware | 34.06 (33.12-35.01) |
| District of Columbia | 18.72 (17.8-19.64) |
| Florida | 24.06 (23.9-24.22) |
| Georgia | 14.94 (14.73-15.15) |
| Hawaii | 18.24 (17.69-18.8) |
| Idaho | 45.04 (44.17-45.92) |
| Illinois | 18.13 (17.94-18.32) |
| Indiana | 33.12 (32.76-33.48) |
| Iowa | 31.41 (30.93-31.89) |
| Kansas | 35.69 (35.13-36.25) |
| Kentucky | 33.4 (32.96-33.83) |
| Louisiana | 23.25 (22.88-23.61) |
| Maine | 29.35 (28.68-30.03) |
| Maryland | 29.96 (29.59-30.33) |
| Massachusetts | 8.02 (7.85-8.19) |
| Michigan | 42.17 (41.86-42.49) |
| Minnesota | 24.09 (23.75-24.43) |
| Mississippi | 8.7 (8.43-8.98) |
| Missouri | 26.3 (25.97-26.62) |
| Montana | 48.35 (47.3-49.39) |
| Nebraska | 38.65 (37.93-39.36) |
| Nevada | 20.21 (19.74-20.67) |
| New Hampshire | 42.88 (42-43.76) |
| New Jersey | 29.41 (29.13-29.69) |
| New Mexico | 31.79 (31.17-32.42) |
| New York | 26.52 (26.33-26.7) |
| North Carolina | 24.4 (24.15-24.66) |
| North Dakota | 62.41 (60.95-63.88) |
| Ohio | 36.72 (36.45-36.99) |
| Oklahoma | 39.12 (38.61-39.63) |
| Oregon | 51.52 (50.96-52.08) |
| Pennsylvania | 24.9 (24.7-25.11) |
| Rhode Island | 35.42 (34.56-36.28) |
| South Carolina | 28.7 (28.31-29.09) |
| South Dakota | 43.09 (41.98-44.19) |
| Tennessee | 27.22 (26.9-27.55) |
| Texas | 34.36 (34.15-34.56) |
| Utah | 22.37 (21.84-22.91) |
| Vermont | 68.81 (67.24-70.39) |
| Virginia | 12.52 (12.32-12.73) |
| Washington | 42.36 (41.95-42.76) |
| West Virginia | 16.02 (15.59-16.45) |
| Wisconsin | 41.53 (41.11-41.94) |
| Wyoming | 49.66 (48.13-51.18) |

CI, Confidence Interval

**Supplemental Table 8:** Age-adjusted mortality rates (AAMRs) for ischemic heart disease mortality with documented tobacco use per 100,000 adults aged ≥ 25 years, stratified by state, in the United States, 2021–2023.

| **State** | **Age Adjusted Rate (95% CI)** |
| --- | --- |
| Alabama | 22.44 (21.61-23.27) |
| Alaska | 38.03 (34.69-41.37) |
| Arizona | 41.45 (40.53-42.38) |
| Arkansas | 38.24 (36.82-39.65) |
| California | 3.23 (3.11-3.34) |
| Colorado | 39.9 (38.79-41.01) |
| Connecticut | 16.21 (15.4-17.02) |
| Delaware | 53.15 (50.41-55.89) |
| District of Columbia | 24.77 (22.08-27.47) |
| Florida | 28.09 (27.68-28.49) |
| Georgia | 19.72 (19.16-20.29) |
| Hawaii | 24.85 (23.3-26.4) |
| Idaho | 45.36 (43.37-47.36) |
| Illinois | 24.51 (23.95-25.06) |
| Indiana | 54.44 (53.3-55.58) |
| Iowa | 58.86 (57.18-60.55) |
| Kansas | 50.24 (48.57-51.91) |
| Kentucky | 60.36 (58.9-61.82) |
| Louisiana | 43.39 (42.41-44.64) |
| Maine | 58.45 (56.09-60.82) |
| Maryland | 28.77 (27.91-29.62) |
| Massachusetts | 22.11 (21.41-22.8) |
| Michigan | 54.49 (53.58-55.4) |
| Minnesota | 49.8 (48.62-50.98) |
| Mississippi | 26.74 (25.53-27.96) |
| Missouri | 46.24 (45.17-47.32) |
| Montana | 57.37 (54.64-60.11) |
| Nebraska | 39.88 (38.06-41.7) |
| Nevada | 44.51 (42.97-46.05) |
| New Hampshire | 52.38 (50.05-54.72) |
| New Jersey | 22.25 (21.64-22.85) |
| New Mexico | 41.45 (39.73-43.17) |
| New York | 28.91 (28.44-29.38) |
| North Carolina | 49.33 (48.47-50.19) |
| North Dakota | 57.96 (54.39-61.52) |
| Ohio | 50.95 (50.13-51.76) |
| Oklahoma | 39.03 (37.75-40.31) |
| Oregon | 53.44 (52.06-54.81) |
| Pennsylvania | 31.39 (30.8-31.97) |
| Rhode Island | 36.74 (34.52-38.97) |
| South Carolina | 32.31 (31.34-33.28) |
| South Dakota | 50.71 (47.67-53.75) |
| Tennessee | 67.93 (66.69-69.18) |
| Texas | 36.15 (35.66-36.64) |
| Utah | 26.9 (25.57-28.24) |
| Vermont | 73.59 (69.61-77.57) |
| Virginia | 35.74 (34.93-36.56) |
| Washington | 49.24 (48.21-50.27) |
| West Virginia | 61.13 (58.94-63.33) |
| Wisconsin | 51.09 (49.94-52.24) |
| Wyoming | 62.58 (58.43-66.73) |

CI, Confidence Interval

**Supplemental Table 9:** Ischemic heart disease mortality with documented tobacco use per 100,000 adults aged ≥ 25 years, stratified by the place of death, in the United States, 1999 to 2023.

| **Place of Death** | **Deaths** |
| --- | --- |
| Medical Facility – Inpatient | 417,904 |
| Medical Facility - Outpatient or ER | 179,648 |
| Medical Facility - Dead on Arrival | 12,839 |
| Medical Facility - Status unknown | 129 |
| Decedent's Home | 602,678 |
| Hospice Facility | 60,982 |
| Nursing Home/Long-term Care | 158,427 |
| Other | 64,013 |
| Place of Death Unknown | 1,299 |
| **Total** | 1,497,919 |

**Supplemental Table 10:** Age-adjusted mortality rates (AAMRs) for ischemic heart disease mortality with documented tobacco use per 100,000 adults aged ≥ 25 years, stratified by urban-rural classification, in the United States, 1999–2020.

| **Year** | **Metropolitan** | **Non-Metropolitan** |
| --- | --- | --- |
| 1999 | 3.56 (3.46-3.65) | 5.42 (5.17-5.68) |
| 2000 | 4.32 (4.22-4.43) | 7.04 (6.75-7.33) |
| 2001 | 4.2 (4.09-4.3) | 7.02 (6.73-7.3) |
| 2002 | 4.27 (4.17-4.38) | 7.3 (7.02-7.59) |
| 2003 | 12.13 (11.96-12.31) | 18.2 (17.74-18.65) |
| 2004 | 16.26 (16.06-16.46) | 24.02 (23.51-24.54) |
| 2005 | 21.04 (20.82-21.27) | 28.33 (27.77-28.89) |
| 2006 | 22.21 (21.98-22.44) | 29.37 (28.81-29.93) |
| 2007 | 23.61 (23.38-23.85) | 31.01 (30.43-31.58) |
| 2008 | 25.62 (25.37-25.86) | 35.95 (35.33-36.56) |
| 2009 | 24.4 (24.16-24.63) | 35.16 (34.56-35.77) |
| 2010 | 26.1 (25.86-26.34) | 39.16 (38.52-39.79) |
| 2011 | 26.81 (26.57-27.05) | 42.17 (41.51-42.82) |
| 2012 | 27.98 (27.74-28.22) | 45.94 (45.25-46.62) |
| 2013 | 28.36 (28.11-28.6) | 46.26 (45.58-46.94) |
| 2014 | 28.33 (28.09-28.57) | 46.91 (46.23-47.59) |
| 2015 | 29.22 (28.98-29.46) | 49.73 (49.03-50.42) |
| 2016 | 29.65 (29.41-29.89) | 49.52 (48.82-50.21) |
| 2017 | 30.03 (29.79-30.26) | 50.66 (49.96-51.36) |
| 2018 | 30.23 (30-30.47) | 51.58 (50.88-52.27) |
| 2019 | 30.05 (29.82-30.28) | 52.01 (51.32-52.71) |
| 2020 | 31.91 (31.68-32.15) | 55.88 (55.16-56.6) |
